# Fast-track breeding system to introduce CTV resistance of trifoliate orange into citrus germplasm, by integrating early flowering transgenic plants with marker-assisted selection

**DOI:** 10.1186/s12870-020-02399-z

**Published:** 2020-05-19

**Authors:** Tomoko Endo, Hiroshi Fujii, Mitsuo Omura, Takehiko Shimada

**Affiliations:** 1 National Agriculture and Food Research Organization, Institute of Fruit and Tea Tree Science (NIFTS), Shizuoka, Shimizu 424-0292 Japan; 2 Faculty of Agriculture, Shizuoka University, Shizuoka, Suruga 422-8529 Japan

**Keywords:** *Poncirus trifoliata*, CTV resistance, Citrus, Juvenile fruit tree, Null segregant, Backcross, Breeding period

## Abstract

**Background:**

Global warming will expand the range of new and invasive pathogens in orchards, and subsequently increase the risk of disease epidemics and economic losses. The development of new resistant plant varieties can help to reduce the impact of pathogens, however, the breeding speed can be extremely slow, due to the growth rates of the plants, and the availability of resistance genes. Citrus trees are suffering immense damage from serious diseases such as citrus canker (XCC), huanglongbing (HLB), and citrus tristeza virus (CTV). A fast-track breeding system, that aimed at shortening the duration for disease resistance breeding by incorporating the resistance genes from related species to commercial varieties, has been developed using the integration of precocious transgenic trifoliate orange with the overexpression of *CiFT* and MAS. It was applied here to incorporate CTV resistance of trifoliate orange into citrus germplasm.

**Results:**

One generation of backcrossed breeding, that would normally take at least 5 years, was achieved in a single year by fast-track breeding system. Linkage analysis using the corresponding DNA markers revealed that CTV resistance and T-DNA integrated regions were found in different linkage groups, and they were independently segregated in the BC progenies. The CTV resistant null segregants, in which the T-DNA integrated region was removed from their genome, were feasibly obtained by MAS in each generation of the BC progenies, and their CTV resistance was confirmed by immunological analysis. Several BC_3_ null segregants, whose genetic backgrounds had been substituted into citrus germplasm, except for the haplotype block of CTV resistance, were successfully obtained. CGH and NGS analyses revealed that the T-DNA integrated region was safely segregated out in null segregants.

**Conclusion:**

Fast-track breeding systems are expected to shorten the required breeding time by more than one-fifth in comparison with conventional cross breeding techniques. Using this system, we obtained BC_3_–8, whose genetic background was successfully substituted except for the CTV resistance locus, and could be a novel mandarin breeding material. The fast-track breeding system will be useful to introduce important traits from related species to citrus germplasm while also drastically reducing the time required for breeding.

## Background

Fruit trees such as citrus, apple, and peach, generally have long breeding periods and the development of new varieties by conventional cross breeding, requires at least a dozen years, if not more. This long breeding period is mainly due to the long juvenile phase of perennial trees; for fruit trees this varies from 3 to 15 years, or more [1]. Recently, the fruit industry has faced inevitable issues resulting from increased global warming, due to climate change. Global warming will increase the risk of epidemics from new and invasive pathogens in orchards. The development of new resistant varieties is one of the key mitigating ways to protect the fruit industry from these upcoming issues. General plant breeding for disease resistance is carried out by conventional breeding, which hybridizes known varieties with sources of resistance, and then selects for resistant lines. However, the time required for the development of a new variety with this method, cannot realistically occur in tandem with the development and spread of new disease epidemics, owing to the time requirement for breeding and the lack of natural resistance resources. Citrus canker caused by *Xanthomonas citri pv. citri* (XCC), huanglongbing (HLB) caused by *Candidatus* Liberibacter, and citrus tristeza virus (CTV), are the three major bacterial and viral diseases that cause grave damage to citrus orchards around the world. There is no effective management strategy for HLB, as sources of resistance are still unknown, and it is recommended that infected trees be removed to prevent its further spread. Kumquat (*Fortunella spp*) and trifoliate orange (*Poncirus trifoliata* L. Raf.), which are closely related to the genus *Citrus* and possess sexual compatibility with general citrus, are known to be highly resistant to XCC and CTV, respectively. Some commercial and conventional varieties have a kind of field resistance against these diseases, but their resistance might be controlled by multiple genes and resistant phenotypes can collapse in breeding populations. Therefore, citrus breeders would like to introduce the genetic resistance of kumquat and trifoliate orange to commercial varieties. However, some of the unfavorable traits, such as low fruit quality (low sugar, high acid, bitter, etc.), small fruit, hard skin, long thorns, and so on, make them hesitant to progress with disease resistance breeding. As citrus trees have a juvenile phase of more than 5 years, it can take approximately 20 years for a new variety to go from development to being cultivated for commercial shipment in Japan [2]. An intergeneric hybrid, ‘US119’, was developed in the USA [3], and ‘Kankitsu Chukanbohon Nou 8 Gou’ (‘Nou-8’) in Japan [4], was released as CTV resistant breeding resources, but both backcrossed strains ‘US119’ and ‘Nou-8’ have unfavorable traits that originate from trifoliate orange, such as high acidity, acridity, and long thorns. Therefore, a further backcross is required to remove these unfavorable traits. Marker-assisted selection (MAS) systems have been introduced to conventional mandarin breeding to improve breeding efficiencies, using DNA markers for CTV resistance [5], male-sterility [6], and monoembryony [7]. MAS could increase the efficiency of the limited field space available for citrus breeding in Japan, by planting seedlings with target traits. However, the reduction of the long breeding period has previously been an issue for which there was no available solution.

Various efforts have been carried out to reduce the long juvenile phase in the past decade, so that both the evaluation of fruit traits and crossbred citrus seed acquisition, can occur earlier. Precocious flowering occurs in the seedlings of pummelo (*Citrus maxima* (Burm.) Merr.) and grapefruit (*C. paradisi* Macf.) [8]. The molecular mechanisms of their precocious phenotype were unclear, and the phenotype was unstable or disappeared, when the genetic background was substituted to the germplasm of the cultivated citrus. This made it hard to utilize this phenotype to reduce the conventional breeding period. The characterization in *Arabidopsis* of various genes regulating flowering and development, resulted in the utilization of several genes to reduce the long juvenile phase in woody perennials over the past decade [9]. In citrus, Peña et al. (2001) succeeded in reducing the generation time of citrange, an intergeneric hybrid between sweet orange (*Citrus sinensis* Osbeck) and trifoliate orange, with the introduction of either *APETALA1* (*AP1*) or *LEAFY* (*LFY*) [10]. The ectopic expression of either gene conferred fertile flowers and fruits on transgenic citrange within one and a half years. These two genes had different effects on the phenotypes of the apomictic or zygotic offspring. They reported that *AP1* was more efficient than *LFY* because *AP1* triggered less abnormality in the vegetative growth than *LFY*. Endo et al. (2005) succeeded in reducing the generation time of trifoliate orange with the introduction of citrus *FLOWERING LOCUS T* (*CiFT*). Endo et al. (2009) developed a *CiFT* co-expression vector system, that drives the target gene with the *CiFT* and the 35S promoter, and succeeded in aroma engineering of trifoliate orange, to reduce the limonene content a few years after *Agrobacterium* inoculation [11, 12]. Velázquez et al. (2016) developed a *Citrus leaf blotch* virus-based vector (*clbvINpr*-*AtFT* and *clbvINpr*-*CiFT*) and citrus plants of different genotypes inoculated with either of these vectors started flowering within 4 to 6 months, with no alterations to the plant architecture, leaf, flower, or fruit morphology, in comparison with the non-inoculated adult plants [13]. Thus, transgenic technologies that utilize the flowering genes, could dramatically speed up genetic studies of fruiting traits.

Recently, several attempts have been reported to reduce the breeding period in fruit trees using the integrated method of early flowering transgenic plants and MAS. In apple (*Malus × domestica*) breeding, transgenic early flowering plants overexpressing a FRUITFULL-homolog (*BpMADS4*) were crossed with T1190 that had a fire blight-resistance gene (Fb-F7) from the wild species of *Malus* [14]. The early flowering transgenic F_1_ seedlings were backcrossed with ‘Regia’, with apple scab resistance genes (*Rvi2* and *RVi4*), and 98/6–10 with powdery mildew resistance genes (*Pl-1* and *Pl-2*), resulting in a plurally resistance gene pyramid, achieved by MAS, in transgenic BC_1_ seedlings. The T-DNA inserted locus could be segregated independently with target genes among these progenies when their genetic loci were different to each other. It was confirmed that the progenies with the T-DNA flowered within 15–40 weeks from planting of the seed. Thereafter, various transgenic lines with different genetic locus of T-DNA insertions harboring *BpMADS4,* were constructed to work compatibly with the various resistance genes, aiming for the selection of non-transgenic null segregants at the end of the rapid cycle of the breeding system [15]. They finally succeeded in generating BC_4_ null progenies with fire blight and apple scab resistance in the green house, after 7 years [16]. In addition, Yamagishi et al. (2011) developed a rapid flowering system in apple based on the *Apple latent spherical virus* vector expressing an *FT* gene of *Arabidopsis thaliana* (*FT-ALSV*) [17]. *FT-ALSV* infected apple seedlings flowered within 2 months, after seed planting, and the next-generation of seeds were obtained within 7 months, when the pollen from the precocious flowers could be used for pollination. Most next-generation seedlings obtained from *FT-ALSV* infected apples were free of viruses and *FT-ALSV* could also be eliminated with high-temperature treatments [18]. These rapid cycle breeding systems maximized the potential of BC breeding to introduce the important genes of wild species into cultivated varieties, through the genetic background substitution with the help of MAS.

Herein, the fast-track breeding system using the integrated method of precocious transgenic trifoliate orange and MAS, was applied to introduce CTV resistance of trifoliate orange into citrus germplasm, as a first attempt to shorten the breeding period of disease resistant varieties. Precocious transgenic trifoliate orange harboring *P35S::CiFT* (T_0_–2-11) [11], and MAS for CTV resistance were used to enhance both of the speed and efficiency in BC breeding. The fast-track breeding system succeeded in promoting one breeding generation in the shortest time, a year in contrast that it would take at least 5 years in general BC breeding. As the genetic loci of CTV resistant and T-DNA integrated regions were different in T_0_–2-11, they were independently segregated in the BC progenies, resulting in CTV resistant null segregants, lacking a T-DNA integrated genomic region, that were feasibly obtained in each generation of the BC population. Comparative genomic hybridization (CGH) and next generation sequencing (NGS) analyses were carried out to confirm the detachability of the T-DNA integrated region in the null segregants. It was demonstrated that precocious transgenic trifoliate orange and the MAS of the target traits were compatible in the fast-track breeding system, when their genetic loci were located on different linkage groups, and the T-DNA integrated region was not retained in the genomes of the null segregant. The validity of the fast-track breeding system and the evaluation method for the retainability of the T-DNA integrated region in the null segregant is also discussed.

## Results

### Pollination of transgenic trifoliate orange with ‘Hyuganatsu’ and Clementine mandarin

**Fig. 1:**
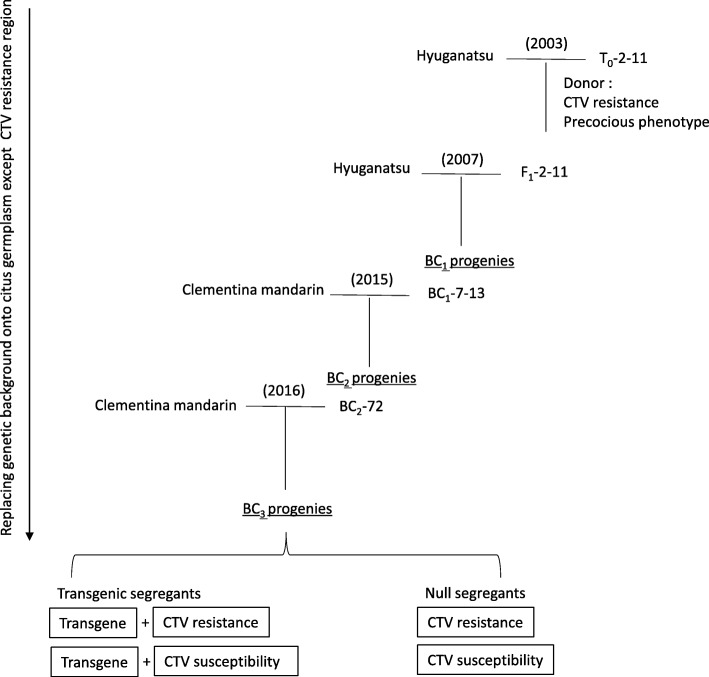
Scheme of fast-track breeding system to introduce CTV resistance into citrus germplasm. Precocious transgenic trifoliate orange that overexpresses *CiFT* (T_0_–2-11) was used as a primary pollen parent for the CTV resistance donor. BC progenies with T-DNA integrated regions generally flower around 3 weeks after planting. F_1_–2-11, BC_1_–7-13, and BC_2_–72 were selected as the pollen parents with DNA markers, to possess CTV resistance and transgenes. In 2016, the flowering times of the seed parent and transgenic seedling were coordinated, to promote the breeding from the BC_2_ to the BC_3_ generation, in a single year

**Fig. 2:**
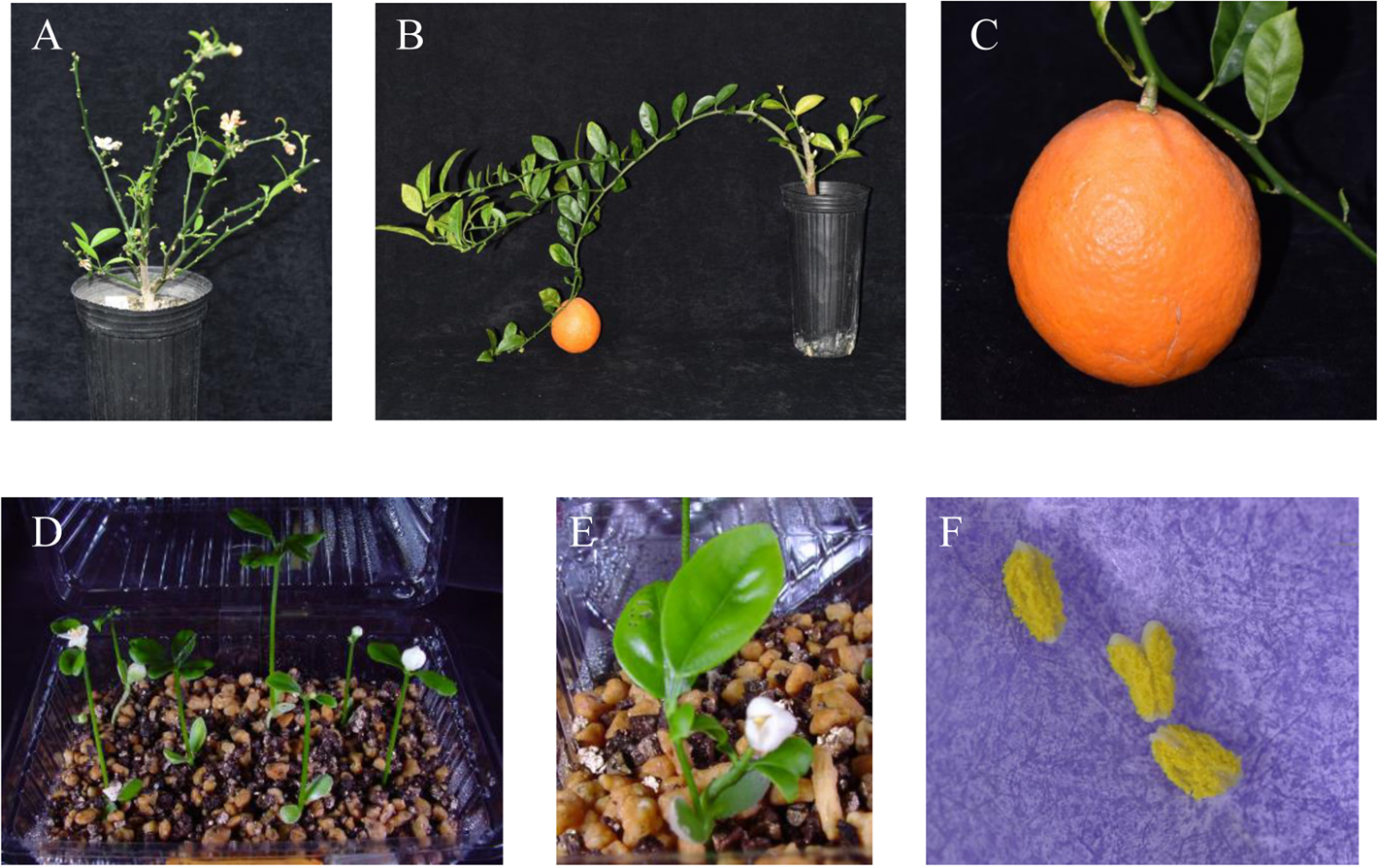
Precocious flowering and fruiting of the BC_2_ progenies **a, b, c** and precocious flowering of the BC_3_ progenies around 3 weeks after seed planting **d**. The normal flower **e** was generated in the BC_3_ progenies with transgenes and fertile pollen **f** that was obtained from the flowers

The transgenic trifoliate orange line (T_0_–2-11), that was molecularly characterized in a previous report [11], was used as a donor of CTV resistance and the precocious phenotype for the fast-track breeding system. T_0_–2-11 possessed only one copy of the T-DNA integrated region harboring *35S::CiFT* in the genome, and it was confirmed by Southern blot analysis in a previous report. The pollen of T_0_–2-11 was crossed with the flowers of ‘Hyuganatsu’ (*C. tamurana* Hort. ex Tanaka) in 2003 and 32 F_1_ seeds were obtained (Fig. 1). The germinated F_1_ progenies were checked for the presence or absence of CTV resistance and for the transgenes in their genomes using the CTV selection markers of CTg06B [5], and a specific designed primer set for the transgene. In 2007, the pollen of F_1_–2-11 harboring both the CTV resistance and the transgene, was crossed with the flowers of ‘Hyuganatsu’, and 136 BC_1_ seeds were obtained. In 2015, the pollen of the BC_1_–7-13, harboring both genes, was crossed with the flowers of Clementine mandarin (*C. clementina* Hort. ex Tanaka), and 576 BC_2_ seeds were obtained. In 2016, the pollen of BC_2_–72 harboring both genes, was crossed with the flowers of Clementine mandarin and 146 BC_3_ seeds were obtained. The precocious phenotype was observed in all the BC progenies harboring the transgene. Their first flowering occurred around 3 weeks after seed planting, but the second flowering varied depending on the individuals in the BC progenies. Flower structures for all of the BC progenies were normal, and their pollen fertility and pollen numbers were sufficient for pollination (Fig. 2); these phenotypical features were similar to those previously reported [11] and were stably inherited to the next generations. Most of the pollinated flowers generally grew to fruits without a severe physiological fruit drop. There were several periods of suspended progress in the fast-track breeding system, due to financial reasons, however, we succeeded in promoting one breeding generation in a single year, from 2015 to 2016.

### Construction of genetic linkage maps using BC_1_ progenies

**Fig. 3:**
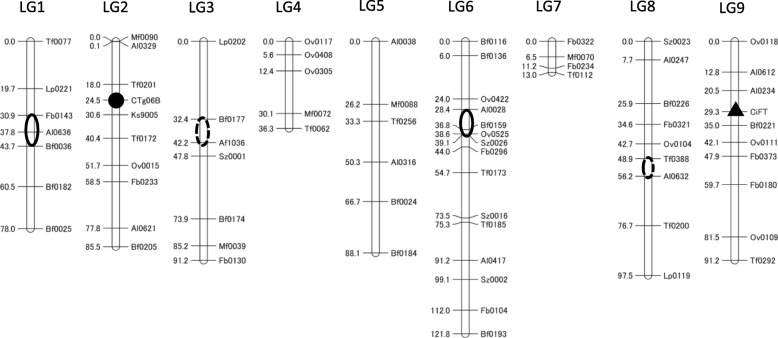
Genetic linkage map using the BC_1_ population obtained from the cross between ‘Hyuganatsu’ and F_1_–2-11. The CTV resistance locus (●) and T-DNA integrated region locus (▲), located on LG2 and LG9, respectively. CAPS markers assigned on the linkage map are utilized for graphical genotyping analysis, for the evaluation of the genetic background substitutions from the trifoliate orange onto the citrus germplasm. The solid ring indicates a QTL controlling a trifoliate leaf, and the hollow black ring indicates a QTL controlling the long thorns

To identify the genetic loci of CTV resistance and the T-DNA integrated region on the genome of T_0_–2-11, a genetic linkage map was constructed using 71 CAPS markers, that were reported in the past genetic map [5, 19, 20], as well as two DNA markers for CTV resistance (Ctg06B) and T-DNA integrated region (*CiFT*). These markers were applied to the genotyping of 125 BC_1_ progenies obtained from the cross between ‘Hyuganatsu’ and F_1_–2-1. CTV resistance in trifoliate orange is controlled by a single dominant gene [21], and the genetic locus controlling CTV resistance (*Ctv*) was molecularly well characterized using linkage analysis and genomic sequencing analysis [5, 22–24]. The genetic linkage map spanned 702.5 cM, with 73 loci, in 9 LGs (Fig. 3). The genetic loci of the CTV resistance and the T-DNA integrated region were assigned between Tf0201 and Ks9005 in LG2 and between Al0234 and Bf0221 in LG9, respectively. They were independently segregated in the BC_1_ progenies, and their segregation ratio was approximately 1:1, like that of single gene inheritance (Table 1). An average of 8.1 CAPS markers per LG were assigned, and these assigned markers were useful to trace the allele substitution in each genetic locus of the following BC progenies. Several unfavorable traits specific to trifoliate orange were also assigned onto the genetic linkage map by quantitative trait locus (QTL) analysis. The QTLs controlling long thorns were mapped between Bf0177 and Af1036 on LG3 (LOD = 1.08), and Tf0388 and Al0632 on LG8 (LOD = 1.12), and they were weak QTLs. The QTLs controlling the trifoliate leaf were mapped between Fb0143 and Bf0036 markers on LG1 (LOD = 1.22), and Al0028 and Sz0026 on LG6 (LOD = 1.32), and they were weak QTLs.

**Table 1:** Segregation of CTV resistance and transgene in BC progenies

Generation	Obtained seeds	Analyzed seeds	CTV (+) Trans gene (+)	CTV (−) Trans gene (+)	CTV (+) Trans gene (−)	CTV (−) Trans gene (−)
F_1_	32	31	7	8	7	9
BC_1_	136	125	19	46	16	44
BC_2_	576	156	46	42	33	35
BC_3_	146	79	22	16	26	15

### Evaluation of genetic background substitution in BC_3_ null segregants with a graphical genotyping map

**Fig. 4:**
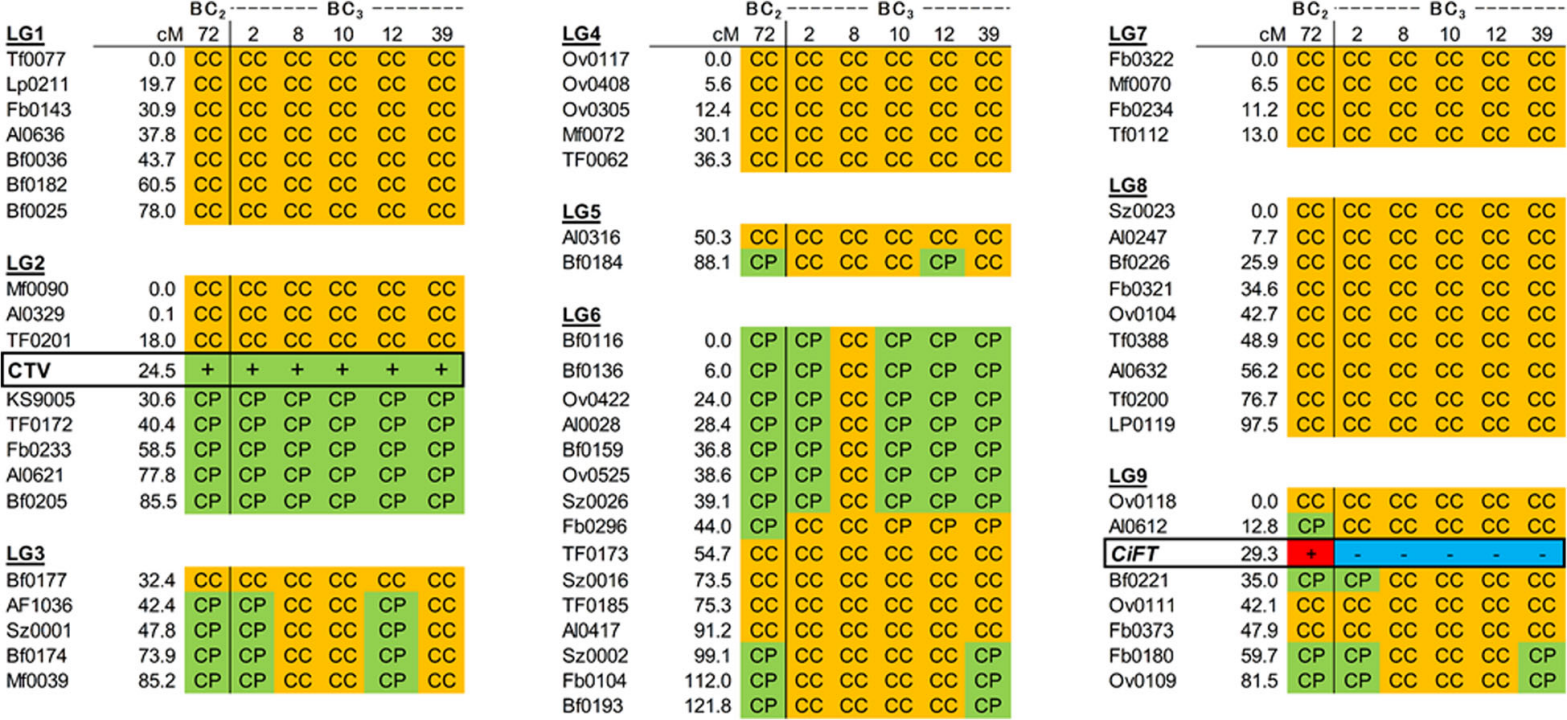
Graphical genotyping map for the evaluation of the genetic background substitutions using representative BC_2_ and BC_3_ progenies. The successful BC_2_ and BC_3_ breeding generations were compared with the citrus germplasm background. BC_3_–8 had successfully substituted its entire genetic background for the citrus germplasm, except for the haplotype block of CTV resistance on LG2. CC: Homogenous locus of citrus genotype. CP: Heterozygous locus of the citrus and trifoliate orange genotypes. +: Target gene is present. -: Target gene is absent

The 61 CAPS markers which could be applicable to trace the alleles derived from trifoliate orange, as well as the DNA selection markers for CTV resistance and the transgene, were applied to the genotyping of the BC_3_ null segregants of BC_3_–2, BC_3_–8, BC_3_–10, BC_3_–12, BC_3_–39, and their pollen parent BC_2_–72. DNA diagnosis enabled us to understand the genetic composition of the BC progenies and rapidly answered the question regarding the presence or absence of the CTV resistance as well as the transgene. In Fig. 4, the genotyping information was summarized as a graphical genotyping map for each LG. In BC_2_–72, the haplotype blocks comprising “P” alleles derived from trifoliate orange were observed in LG2, LG3, LG5, LG6, and LG9. The genetic background of LG1, LG4, LG7, and LG8 had already been substituted into “C” alleles derived from the citrus germplasm. During the development of BC from its BC_2_ generation to its BC_3_ generation, these haplotype blocks of trifoliate orange were randomly segregated out from those LGs and were substituted with those of citrus. For the QTLs controlling long thorns derived from trifoliate orange, the pollen parent of BC_2_–72 already lacked a QTL on LG8, but still possessed a part of the QTL on LG3, where recombination between the trifoliate orange and citrus haplotypes occurred. This remaining QTL on LG3 was thereafter segregated out in some of the BC_3_ progenies. For the QTL controlling the trifoliate leaf, the pollen parent of BC_2_–72 already lacked a QTL on LG1, but still possessed a QTL on LG6. This remaining QTL on LG6 was segregated out in some BC_3_ progenies, but it remained in most of the examined BC_3_ progenies. Those phenotypes almost disappeared in the BC_2_ and BC_3_ progenies.

The genetic background of the BC_3_–8 was successfully substituted for all the LGs, except for LG2 where the CTV resistance locus was located. Although one more generation would be required to completely substitute the remaining haplotype block of the trifoliate orange, BC_3_–8 could be a new mandarin breeding material with CTV resistance. Thus, several unfavorable traits of trifoliate orange could be also removed to progress the breeding generation with this fast-track breeding system. These results revealed that it would likely take at least 4 generations or more to substitute the genetic background and to import the target gene from the related species, such as the trifoliate orange and kumquat to citrus germplasm.

### CGH analysis to detect the vector construct sequence in the BC progenies

**Fig. 5:**
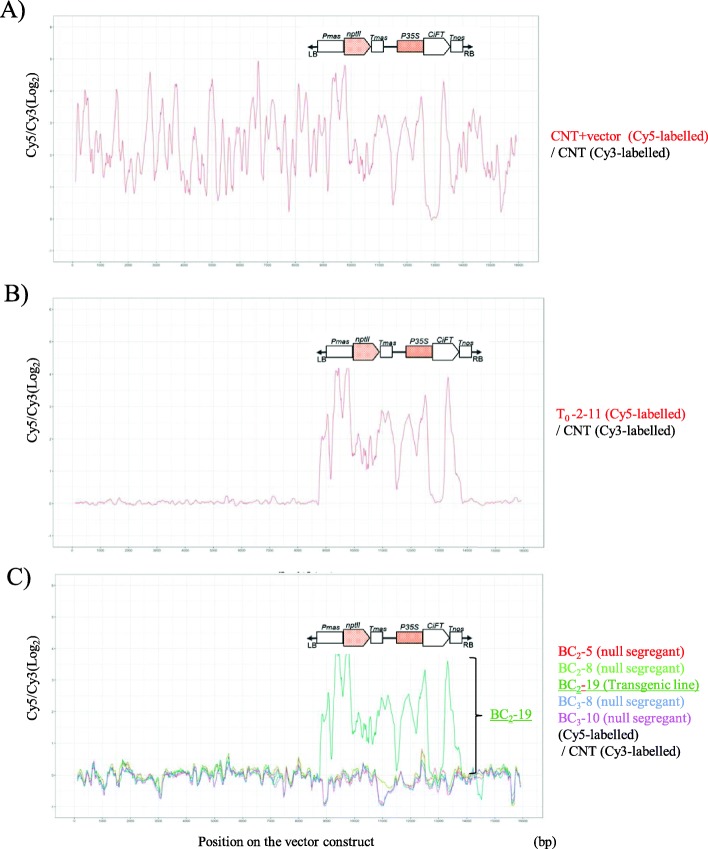
CGH analysis to detect the sequence of the T-DNA insertion regions for the vector construct generated from the pCGN157 in the BC progenies. Genomic DNA of non-transgenic trifoliate orange (CNT) labeled with Cy3 is used for basic control sample. Target samples are labelled with Cy5. The signal intensity is revealed by the log_2_ ratio of Cy5/Cy3 using a moving average of 20 adjacent 60-mer oligonucleotide probes

A custom CGH array comprising 12,736 of 60-mer probes, was designed based on the 15,920 bps of the nucleotide sequence for the vector construct generated from the pCGN157 binary vector [11]. The Cyanine 5 (Cy5)-labelled mixture of genomic DNA of the control non-transgenic trifoliate orange (CNT) and vector constructs, T_0_–2-11, BC_2_–5, BC_2_–8, BC_2_–19, BC_3_–8, and BC_3_–10 were hybridized with Cyanine 3 (Cy3)-labelled control genomic DNA of the CNT. To visualize the CGH analysis data, the log_2_ ratio of Cy5/Cy3 for the probes was mapped according to their position on the vector construct. For the hybridization between the Cy5-labelled CNT and the vector construct mixtures and the Cy3-labelled control, the signals were detected from almost all the probes (Fig. 5a). The signal pattern went up and down throughout the whole vector construct, while, the signal was not detected around a part of the *CiFT* cDNA insertion region in the vector construct. The low and missing signal detection might come from inadequate probes, sequences with low GC content and repeated sequences, labeling efficiency to the genome sample, and so on. The missing signal around the *CiFT* cDNA insertion region might have occurred from the interference of the genomic fragment of the endogenous *CiFT.* In contrast, the hybridization between T_0_–2-11 and the Cy3-labelled control revealed that the signals were only detected in the T-DNA region of the vector construct, although there was also a missing signal region around the *CiFT* cDNA insertion region (Fig. 5b). The up and down pattern of the signal intensity patterns around the T-DNA region were almost identical between the control experiments, revealing that the detecting signal pattern was reproducible. Figure 5c showed the hybridization data of the Cy5-labelled BC_2_–5, BC_2_–8, BC_2_–19, BC_3_–8, and BC_3_–10 with the Cy3-labelled control. BC_2_–19 was the transgenic line with the T-DNA integrated region and the similar signal pattern was observed with those of T_0_–2-11. In contrast, the steady signal was not detected in the remaining four null segregants lacking the T-DNA integrated region (BC_2_–5, BC_2_–8, BC_3_–8 and BC_3_–10). Thus, CGH analysis worked for the detection of the T-DNA integrated region derived from T_0_–2-11 in the BC progenies, and demonstrated that the T-DNA integrated region was absent from the genomes of BC_2_ and BC_3_, the null segregants.

### NGS analysis to detect the vector construct sequence in the BC progenies

**Fig. 6:**
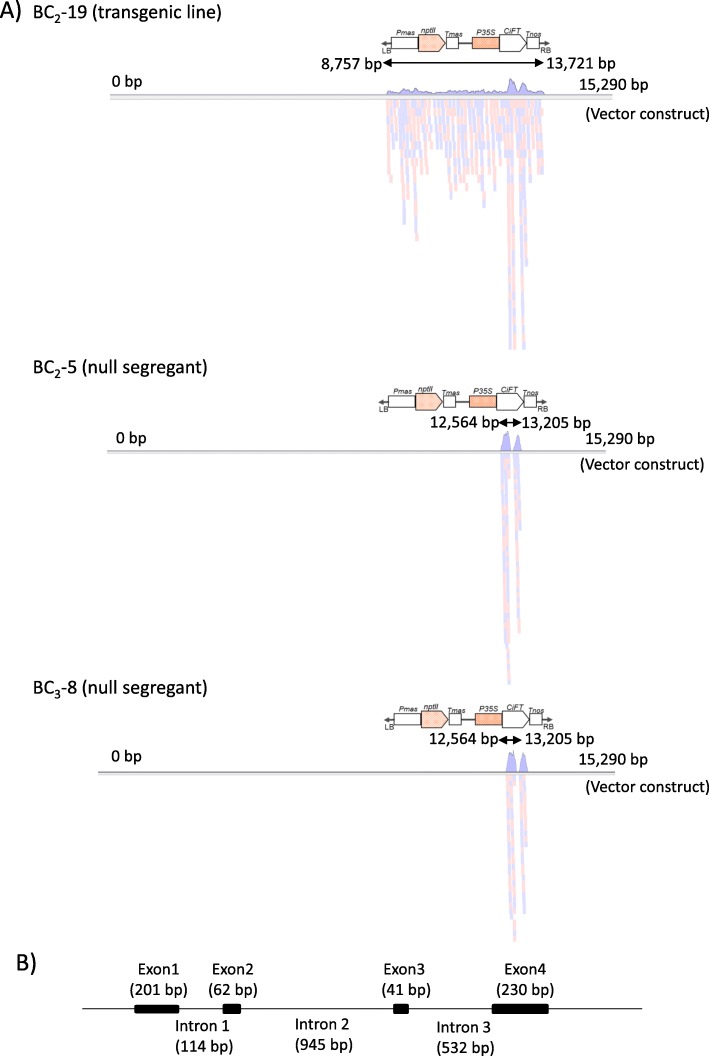
NGS sequence assembly of the vector construct **a** and genomic structure of *CiFT* (Ciclev10012905m) **b**. The paired-end reads are assigned onto the whole vector construct generated from the pCGN157 in the BC_2_–19 with the T-DNA integrated region, while the paired-end read sequences were only assigned around the *CiFT* cDNA insertion region, on the vector constructs in BC_2_–5 and BC_3_–8. The genomic sequence of *CiFT* showed that intron 2 had a longer length, compared with exons 2 and 3, and this structure would hamper the sequence assignment around the *CiFT* cDNA insertion regions of exons 2 and 3, in the vector construct of the null segregates

NGS analysis was carried out as an alternative method to detect the vector construct sequence in the BC_2_–5 (null segregant), BC_2_–19 (transgenic line), and BC_3_–8 (null segregant). A total of 4503 Mb, 4802 Mb, and 4746 Mb of Illumina paired-end reads were obtained, respectively, which was more than a 15 × coverage of the assembled genome sizes of the *C. clementina* (301 Mb) [25]. These sequences were assigned to the vector construct sequence with the program of Genetyx-Genome (Software Corporation, Tokyo, Japan) (Fig. 6a). The vector construct had 15,920 bp sequences, comprising the T-DNA region located from 8726 bp to 13,741 bp, with a 25 bp border repeat sequence at both ends, and the *CiFT* insertion from 12,556 bp to 13,265 bp. The paired-end read sequences of the BC_2_–19 were assigned from 8757 bp to 13,721 bp of the vector construct, and corresponded to sequence within the T-DNA region of the vector construct. The sequence assignment depth was almost uniform throughout the vector construct, except for the *CiFT* cDNA insertion region. The assignment depth around this region was extraordinarily high, when compared to the other vector regions. This is explained by the cross assignment with the endogenous FT genomic sequences of ‘Hyganatsu’, Clementine mandarin, or trifoliate orange in the BC progenies. In fact, several SNPs were detected in the *CiFT* cDNA insertion region. Based on the adjacent sequences around the T-DNA integrated site, it was found that T-DNA was inserted onto upstream region of P_trifoliata_00538:mRNA_10.1(Histone H2B protein) at the position between 83,134 bp and 83,146 bp of P_trifoliata_00538 scaffold of trifolate orange genome assembles in the Mikan Genome Database (MiGD: https://mikan.dna.affrc.go.jp/) (Supplementary Table 1 and Fig. S1). The paired-end read sequences of the BC_2_–5 and BC_3_–8 were only assigned to the *CiFT* cDNA insertion region from the 12,564 bp to 13,205 bp region. The unassigned regions in the BC_2_–5 and BC_3_–8 were around where the long length intron 2 of *CiFT* was sandwiched by the short lengths of exon 2 and exon3 (Fig. 6b). Thus, the obtained results from the NGS analysis revealed that the null segregants did not possess any vector construct sequences, and agreed with those in the CGH analysis.

### Evaluation of CTV resistance of *BC*_*2*_*null segregants by* immunological detection

**Fig. 7:**
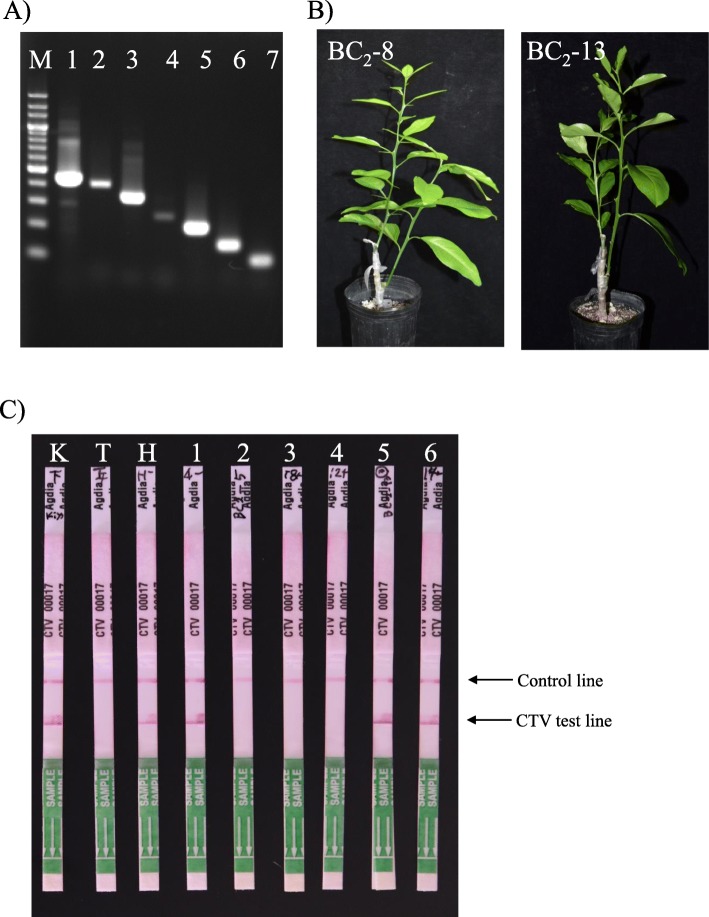
Evaluation for CTV resistance of null segregates, using ImmunoStrip® for citrus tristeza virus (CTV) complete kit (Agdia). **a** PCR fragment amplified from five CTV lines, which have already infected in mother tree of ‘Kiyomi’ by specific primers, reported by Roy et al. (2010). M: 200 bp ladder, 1: EF1-α (884 bp), 2: T36 (836 bp), 3: CPG (positive control: 672 bp), 4: B165 (510 bp), 5: T3 (409 bp), 6: VT (302 bp), 7: T30 (206 bp). **b**) Photograph of rough lemon trees grafted with BC_2_–8 (left) and BC_2_–13 (right) scions on the top, with side a grafting of the CTV infected-mother tree, ‘Kiyomi scion. **c** Immunological detection of CTV. The presence of CTV test lines indicates that CTV propagation has occurred in the shoot and the absence the test lines indicates that CTV propagation was suppressed. K: mother tree of ‘Kiyomi’ scion, T: Trifoliate orange scion (CTV resistant), H: ‘Hyuganastu’ scion (CTV susceptible), 1: BC_2_–4 scion (CTV susceptible), 2: BC_2_–5 scion (CTV resistant), 3: BC_2_–8 scion (CTV resistant), 3: BC_2_–12 scion (CTV resistant), 5: BC_2_–13 scion (CTV susceptible), 6: BC_2_–14 scion (CTV susceptible)

CTV resistance from the trifoliate orange was introduced into the citrus germplasm with the fast-track breeding system. It is important to confirm that CTV-marker selected individuals should be resistant to CTV phenotypically also in null segregants of BC progenies. To evaluate the actual CTV resistance, immunological analysis was carried out on CTV-inoculated null segregants using ImmunoStrip® for Citrus tristeza virus (CTV) Complete kit (Agdia). As the growth of the BC_3_ null segregants with CTV resistance were not enough to prepare the scion for grafting, the scions were prepared from six BC_2_ null segregants and were grafted on the top of the CTV-free 8-month-old rough lemon (*C. jambhiri* Lush) seedlings. The scion of the CTV infected-mother tree ‘Kiyomi’, preserved in NIFTS, was also grafted onto the middle. The scions of the trifoliate orange and ‘Hyuganatsu’ were also grafted as resistant and susceptible controls, respectively. The genotype of the CTV line that had already infected the mother tree ‘Kiyomi’, were investigated with the reverse-transcription polymerase chain reaction (RT-PCR) technology reported by Roy et al. (2010) [26]. PCR fragments corresponding to T36 (accession number: U16304), B165 (accession number: EU076703), T3 (accession number: EU857538), VT (accession number: U56902), and T30 (accession number: AF260651) were detected in RT-PCR using a template cDNA generated from the young shoot of the mother tree ‘Kiyomi’, as well as the control fragment of EF1-α and CPG (Fig. 7a). The CPG were designed to detect the common region among the examined five CTV lines and could detect all CTV lines, regardless of the identifiable or unidentified CTV genotypes [26]. Although the fragment abundance was different among the detected CTV lines, it was found that ‘Kiyomi’ mother tree was infected by the above five CTV lines. Two months after grafting, the leaves of the scions were sampled and were subjected to immunological analysis (Fig. 7b). The CTV virus was detected in all the leaves from the ‘Kiyomi’ scions that had been grafted onto the middle of the root stock. The CTV was also detected in the leaves of the CTV susceptible ‘Hyuganatsu’ scions, but it was not detected in the leaves of the CTV resistant trifoliate orange scion. In the BC_2_ null segregants, the CTV virus was detected in the leaves of the BC_2_–4, BC_2_–13, and BC_2_–14 scions that were judged to be CTV susceptible null segregants from their DNA analysis. In contrast, there was no CTV virus detected in the leaves of the BC_2_–5, BC_2_–8, and BC_2_–12 scions, that had been determined from DNA analysis to be CTV resistant null segregants. Thus, it was confirmed that the CTV selection markers of CTg06B [5] was extremely efficient at selecting CTV resistant BC progenies.

## Discussion

### The fast-track breeding system drastically reduced the period required for BC breeding

High levels of genomic heterozygosity and long durations of juvenility are the major obstacles to reducing the efficiency of conventional citrus cross breeding. Citrus genomes possess a natural system to maintain high heterozygosity by nucellar polyembryony, sexual incompatibility, and male or female sterility. The most economically important citrus varieties originated from the repeated natural interspecific hybridization of four ancestral taxa (*C. reticulata* Blanco, *C. maxima* Merr., *C. medica* L., and *C. micrantha* Wester) and possess complex interspecific mosaic genomes [27]. The ability of modern breeding to utilize these conventional varieties is hampered by their complex heterozygous genomic structures, and because the typical phenotypes observed in the conventional varieties are frequently broken in their progenies by admixtures of genomes from sexual hybridization [28]. These observations are also applicable to the Japanese breeding program, as the present economic citrus varieties and breeding lines were originally derived from the limited genetic resources of 14 ancestral citrus varieties, by pedigree analysis [29]. Therefore, the most desirable phenotypic traits in the general economic varieties do not come from a specific gene of a specific variety, but arise from desirable allelic and haplotype combinations that were commonly possessed in the ancestral varieties. The present breeding population in Japan is approximately the 5th generation after being crossed with its ancestral varieties. Most of the present breeding population expresses many commercially important traits, such as having high sugar and low acid content, good color and flavor, large fruit size, field resistance to disease, and so on, and implicating that the ideal combination of alleles or haplotype blocks could be arranged and fixed in the genetic loci to control these traits. If related species are crossed with the present breeding population, it will take an extraordinary period to restore the potential of the BC progenies back to the present level. Along with the progress of global warming issues, serious damages caused by XCC, HLB, and so on have become more pronounced in citrus orchards. The development of a new variety with genetic resistance to those serious diseases is highly desirable, but first requires an extraordinary period of BC breeding to promote and develop disease resistance breeding systems.

In this study, it was demonstrated that the developed fast-track breeding system could drastically shorten the breeding period by overcoming the long juvenility in the citrus breeding. Although the experiments were tentatively suspended owing to budget problems, they succeeded in promoting one generation within a year. Among the various flowering genes involved in floral induction in citrus, such as *AP1*, *LFY*, *TERMINAL FLOWER*, *CiFT AGAMOUS-LOKE 24* type MADS-box gene, etc. [10, 11, 30, 31], *CiFT* was confirmed to work well in the fast-track breeding system. As the FT orthologs have conserved and important roles in the induction of flowering in highly divergent species of angiosperms [32], the FT homologs have been frequently utilized to cause precocious phenotypes in various plants [33]. Overexpression of the *CiFT* resulted in the precocious phenotype and normal flowers, with fertile pollens in the BC progenies. This precocious phenotype was stably inherited in the BC progenies even when the genetic background was substituted onto citrus germplasm. BC progenies with transgenes developed flowers around 3 weeks after planting the seed. Therefore, the coordination of flowering period between the seed parent and the pollen parent is very important to ensure the achievement of a one generation promotion in a year. After the first flowering in the BC progenies with transgenes, the next flowering time could not be predicted, particularly if their genetic backgrounds were substituted onto citrus germplasm. In the 2015 pollination, the BC_2_ seeds were planted onto sterile soil around 2 months before the pollination. Then, the pollens were collected from the first flower and were stored in the refrigerator. Before the flowering of the seed parent, candidate pollen parents could be selected by DNA diagnosis. The T-DNA integrated genomic region in the BC progenies could feasibly be removed from their genomes in the process of chromosome segregation. In apples, BC_4_ null progenies with fire blight and apple scab resistance were obtained within 7 years, which was 4–5 times faster than conventional breeding [16]. Considering that citrus trees on average require a 5–10 years juvenility phase [1], the fast-track breeding system is expected to reduce the breeding period at least five-fold.

### MAS play a critical role of assistance to the fast-track breeding system

MAS plays a critical role in increasing the efficiency of the selection of the BC progenies, with or without CTV resistance and T-DNA insertions. If the CTV selection marker was not available, then the one generation promotion a year could not be achieved because the candidate pollen parents could not be selected in the limited amount of time available. In this study, ‘Hyuganatsu’ and Clementine mandarin were used as recurrent parent to remove the unfavorable traits of trifoliate orange in the fast-track breeding system. Graphical genotyping maps revealed that it took four generations from the first cross to substitute all chromosomes from the trifoliate orange type into the citrus type, except that the chromosome where the CTV resistance was located. In fact, the genetic locus around the weak QTLs for long thorns and trifoliate leaf were substituted into the citrus genotypes and these unfavorable phenotypes almost disappeared in the BC_2_ and BC_3_ progenies. The period required for the genetic background substitution was almost similar with natural BC breeding [19], so the BC_2_ progenies did not have full substitution on all chromosomes. They reported that 64% of chromosomes had at least one recombination site and 36% of chromosomes had no recombination site in the BC_2_ progenies, revealing that the low frequency of chromosome recombination was one of the factors that prolonged the period in BC breeding. The *Ctv* locus was physically mapped using a *P. trifoliata* bacterial artificial chromosome library and 282-kb genomic region surrounding *Ctv* locus was sequenced [24]. CTV selection markers of CTg06B was developed referencing to this sequence and the effectiveness of selection using DNA markers has been confirmed in this study. The increased marker density in *Ctv* locus would allow selection for a smaller segmentation during further backcrossing. The target trait of CTV resistance is controlled by a single locus. Therefore, the segregation pattern of the target traits and precocious phenotype was simple in each generation of the BC progenies. The average segregation ratio among the BC progenies was roughly fitted to a 1:1:1:1 of Mendelian fashion for transgenic segregant with CTV resistance: transgenic sergeant without CTV resistance: null segregant with CTV resistance: null segregant without CTV resistance. Therefore, about 25% of progenies could theoretically become the candidate final products. However, if target traits are controlled by multiple genes that are scattered across plural chromosomes, the ratio to obtain the final product drastically goes down. Therefore, larger population sizes, high accuracy of linkage markers, and a series of precocious transgenic lines harboring a single T-DNA integration with different locus, are required to obtain the final product. Our developed DNA markers such as numerous CAPS markers (https://mikan.dna.affrc.go.jp/) and P/M markers [7] would help graphical genotyping for the evaluation of chromosome substitution and the selection of monoembryonic BC progeny to obtain zygotic embryos from sexual crosses. Trifoliate orange and kumquat are known to possess resistance to various diseases such as phytophthora, CTV, nematode, XCC, and so on. Molecular elucidation of these disease resistances and the generation of their DNA selection markers would be indispensable for the promotion of disease resistance breeding using fast-track breeding system.

### Evaluation of the detachability of integrated T-DNA insertions in null segregants by CGH and NGS analyses

The precocious phenotype donor, T_0_–2-11, possessed one copy of T-DNA integration into the genome [11]. The T-DNA integrated region was located on LG9 in the genetic linkage map using the progenies between ‘Hyuganatsu and F_1_–2-1. Considering the segregation ratio was fit to 1:1 in the progenies of each BC generation, the T-DNA integrated region was maintained as a single locus in these BC progenies. In this study, two technically different analyses with different principles for detecting the target sequences were applied to evaluate the separability of the T-DNA insertions in the BC null segregants. In CGH analysis, 60-mer oligonucleotide probes were designed from both strands of the vector contract sequences by overlapping with 5 bp slides and those probes equally covered the whole vector sequences, a total of 24 times. Vector sequences were successfully detected on the sample from a mixture of vector and CNT, T_0_–2-11, and BC_2_–19, although the signal intensities among those probes varied depending on the vector region. Signal intensity differences were likely generated by technical issues because probes were forcibly designed to tile the whole vector sequence and the sequence specificity and melting temperature of the probes could not be equalized. This signaling profile was commonly observed in the past report using genome tiling array [34]. NGS sequences were assigned throughout the vector sequences, except for the *CiFT* cDNA insertion region. The high depth of sequence assignment around the *CiFT* insertion derived from the endogenous *CiFT* genes in the BC progenies as it is known that the sequence and intron-exon structures of the *CiFT* are conservative among citrus and related species. *CiFT* consists of 4 exons and 3 introns in characterized citrus species, of which there are short sequence lengths for the 2nd and 3rd exons and long sequence lengths for the 1st and 2nd introns. Any sequence was not assigned around the 2nd and 3rd exons in the null segregants of the BC_2_–5 and BC_3_–5, because of sequence similarity was low, between cDNA and genome sequences around 2nd and 3rd exons. While, in BC_2_–19, the sequences that would come from the T-DNA integrated region could be successfully assigned to the 2nd and 3rd exons of the *CiFT* cDNA insertion regions in the vector construct. Therefore, NGS analysis correctly detected the differences of the assembled sequences between the null segregant lines and the transgenic lines. We tried to carry out 10 × coverage with the whole genome sequencing for the evaluation, and a similar depth of sequences were at least assigned on the target vector sequences out of the 15 × coverage data. Thus, both analyses successfully worked to detect the target sequences of the T-DNA integrated regions in the genome. NGS analysis could provide more detailed information in the sequence level than the CGH analysis. In fact, the position of T-DNA integrated site on trifoliate orange genome was identified using the adjacent sequences around the T-DNA integrated site. Both analyses demonstrated that the T-DNA integrated regions were not retained in the genomes of the examined null segregants.

## Conclusions

The developed fast-track breeding system can drastically shorten the breeding period by overcoming the long juvenility in citrus breeding. It is expected to reduce the required time by at least five-fold, in comparison with conventional cross breeding. Using this system, we obtained BC_3_–8, of which the genetic background was successfully substituted completely in all LGs, except for LG2, on which the CTV resistance locus was located. BC_3_–8 would be a novel mandarin breeding resource with CTV resistance because the T-DNA integrated region was not detected by the technically different methods of CGH and NGS analyses. We consider that the developed system could be applied to the other targets of the breeding program once DNA selection markers are developed. Recently, various new breeding technologies (NBT) such as the transcription activator-like effector nuclease (TALEN) [35] and the clustered regularly interspaced short palindromic repeats associated the Cas9 (CRISPR/Cas9) system [36], have been developed to enable more rapid genetic modifications. Those technologies require the target gene sequence information, but most agronomical important traits including CTV resistance are not addressed to their causative genes. The developed fast-track breeding system has an advantage against these new technologies so that whole genomic region responsible for the target trait could be incorporated through chromosome recombination using DNA markers. The genetically modified crops generated by the NBTs are frequently discussed regarding the necessity of regulatory authority for genetically modified (GM) crops for food safety and security. An important scientific difference between GM and non-GM depends on the retention of the T-DNA integrated region in the genome. Therefore, the CGH and NGS analyses, as well as the bioinformatic analysis could provide clear information regarding this point. Most commercialized non-GM crops in the word have been developed with methods in which transgenic technology is tentatively used in the generating process but the T-DNA integrated region could be finally removed during chromosome segregation. We hope that our report contributes information to the discussion of the regulatory authority of NBT and that the fast-track breeding system will contribute to reduce the time required for disease resistance breeding, by incorporating important genes of related species into citrus germplasm.

## Methods

### Plant materials

Transgenic trifoliate orange harboring *P35S::CiFT* line No. 2–11 (T_0_–2-11) [11], which was molecularly characterized in a previous report [11], was used as a donor of CTV resistance and precocious phenotype. ‘Hyuganatsu’ or Clementine mandarin (Japanese Genebank registration number: JP113161) were used as the mother plants for BC breeding to substitute the genetic backgrounds from trifoliate orange to citrus germplasm, except for the CTV resistance locus (Fig. 1). The seed parent was switched to prevent the inbreeding depression in the BC progenies. The pollens of T_0_–2-11 and its progenies with the transgenes were collected and stored at − 25 °C until use. Seeds obtained by crossing were germinated and grown as previously described [11]. All materials used in this study were cultivated at the National agriculture and food research organization Institute of Fruit and tea Tree Science (NIFTS), in Okitsu Shizuoka, Japan.

### DNA extraction and construction of genetic map

Genomic DNA was extracted from the fresh and fully expanded leaves of material plants, according to the method described by Dellaporta et al. (1983) [37]. The 125 BC_1_ progenies between ‘Hyuganatsu’ and T_0_–2-11 were subjected to linkage analysis to identify the genetic locus of the CTV resistance gene and the transgene insertion site. The CTV resistance locus was detected using the past reported DNA marker of Ctg06B [5] as follows; forward primer of Ctg06B: 5`-ccaccagtgcccatctacct-3`, reverse primer of Ctg06B: 5`-gacgcctccttgttgacgata-3`. The transgene was detected using following primer set: forward primer for transgene: 5`-atctccactgacgtaagggatgacg-3`, reverse primer for CTV resistance gene: 5`-acaggattcaatcttaagaaactt-3`. A total of 71 CAPS markers were reported in the trifoliate orange genetic map [19], and were applied to construct a genetic map of the BC_1_ progenies. CAPS marker analysis was carried out according the past reported conditions [20] with modification of the restriction enzymes (Supplementary Table 1). Linkage analysis was carried out using the BC_1_ mode of the software, JoinMap ver. 5.0 (Kyazma, Wageningen, The Netherlands). The QTL analysis was performed with MapQTL ver. 6.0 (Kyazma,) for the percentage of occurrence of the trifoliate compound leaf and long thorns. As the length of long thorn phenotype was influenced by overexpression of *CiFT,* sixty BC_1_ null segregants without transgene were used for QTL mapping. The interval mapping was applied to detect the QTLs for the traits of long thorns and trifoliate leaf with the Kruskal-Wallis (KW) test. QTLs with the mean values for LOD > 1.0 were considered as significant.

### Evaluation of genetic background substitution in BC_2_ and BC_3_ progenies by CAPS markers

The genetic background substitution of BC_2_ and BC_3_ progenies was evaluated using the graphical genotyping map. CAPS analysis was carried out to identify the genotype on each genetic locus of the 61 CAPS markers assigned in the above genetic map. The presence or absence of the CTV gene and the transgene were investigated using the above primer sets. The graphical genotyping map was constructed using the genotyping data for 9 linkage groups. The genetic background substitution in BC_2_–72, BC_3_–2, BC_3_–8, BC_3_–10, BC_3_–12, BC_3_–39 were representatively summarized.

### Comparative genome hybridization (CGH) analysis to detect the vector construct sequence in BC progenies

A custom CGH array was designed for the detection of the vector construct sequence in the BC progenies, that was used to generate the T_0_–2-11 of the transgenic parent plants with pCGN1547 as a backbone [11]. Using the web application of SureDesign (Agilent Technologies, Santa Clara, CA, USA; https://earray.chem.agilent.com/suredesign/), 60-mer oligonucleotide probes were designed from both strands of the vector construct sequences by overlapping with 5 bp slides and 3184 probes were designed for each strand. Those probes were mounted in duplicate on the custom oligo-DNA microarray with control probes supplied from Agilent Technologies (Agilent Design number: Agilent-085042) under the 8 × 15 K format of the Agilent system. All array hybridization procedures were in accordance with the manufacture’s recommendations. 350 ng of genomic DNA was digested with *Alu*I and *Rsa*I, labeled with Cyanine 3 (Cy3) or Cyanine 5 (Cy5) with SureTag Complete DNA Labeling Kit (Agilent Technologies). Cy5-labeled DNAs were as follows, array no.1: non-transgenic control trifoliate orange (CNT), no. 2: CNT with the vector construct; no.3, T_0_–2-11, no.4: BC_2_–5, no.5: BC_2_–8, no.6: BC_2_–19, no.7: BC_3_–8, no.8: BC_3_–10. CNT DNA was labeled with Cy3 as an internal control for all arrays. The labeled DNAs were purified with a Purification Column (Agilent Technologies) and absorbance was measured with NanoDrop 1000 (Thermo Fisher Scientific, Waltham, MA, USA) using Microarray Measurement mode. Mixture of Cy5- and Cy3-labeled DNAs were used for array hybridization at 65 °C for 24 h. Standard washing conditions were performed. Scanning conditions of the array were at 5 m resolution and TIFF 20 bit. Image analysis was performed with Agilent Feature Extraction version 12.0.3.1 software. Data were analyzed with GeneSpring GX ver.14.9 software (Agilent Technologies). In order to remove systemic biases among each array, normalization was performed by dividing the Cy5/Cy3 values of the probes by the median of 13 positive control probes, Dark Corner, SM_01 ~ SM_12 in each array. The normalized value of each probe in arrays no. 2 to 8 was divided with the value in arrays No. 1, the negative controls, to remove the effect of nonspecific hybridization to each probe. Sample values of each probe were calculated as log2 ratio, i.e. log_2_ (sample (Cy5/Cy3)/ negative control (Cy5/Cy3)). Probe quality was evaluated based on the flag description in the Agilent Feature Extraction Software 12.0.3.1. Probes with the flag ‘Not Detected’ in all 8 arrays were excluded from the analysis. Sample log_2_ values were used to calculate the moving average of 20 probes. The complete microarray data have been deposited in the NCBI Gene Expression Omnibus (http://www.ncbi.nlm.nih.gov/gds) series entry GSE135027.

### NGS analysis to detect the vector sequence in the BC progenies

Genomic DNA of BC_2_–5, BC_2_–19 and BC_3_–8 were utilized to construct Illumina paired-end (PE) libraries with the average insert size of 350 bps using the TruSeq DNA PCR-Free Library Preparation Kit. Each library was sequenced using a one-lane flow cell in the Illumina HiSeq 2000 system with a read length of 100 bp. Low-quality bases and Illumina sequencing adapters were trimmed using Trimmomatic v.0.36 (ILLUMINACLIP: TruSeq3-PE-2.fa:2:30:10 LEADING:20 TRAILING:20 SLIDINGWINDOW:10:15 MINLEN:50) and NxTrim (−-minlength 50) for Illumina PE reads [38]. The Illumina PE reads were aligned with the referencing sequences of vector construct [11] using the sequencing assembling program of Genetyx-Genome ver. 1.1.1 in the computer software Genetyx-Win Ver. 13.0 (Software Development). BC_2_–5 and BC_3_–8 were null segregants lacking transgene, and BC_2_–19 was transgenic segregant with the transgene inserted in the genome.

### Evaluation of CTV resistance of BC_2_ null segregants by immunological detection

The scions of six BC_2_ null segregants with or without CTV resistance gene were grafted on the top of 8-month-old seedlings of Rough lemon, in which the scion of CTV infected-mother tree of ‘Kiyomi’ preserved in NIFTS was grafted on the middle. The genotype of CTV infecting the mother tree ‘Kiyomi’ was identified using multiplex reverse-transcription polymerase chain reaction (RT-PCR) technology reported by Roy et al. (2010) [26]. Elongation factor 1 alpha (EF1-α) was used as endogenous transcription control in the mother tree ‘Kiyomi’ and the PCR fragments were amplified with the following primer set: forward primer, 5`- aaggctgagcgtgaacgtgg-3` and reverse primer, 5`-acggcaatgtgggaggtgtg-3`. Total RNA was extracted from the fresh leaves using RNeasy® plant Mini Kit (Qiagen, Hilden, Germany), and the cDNA was prepared with 1 μg of purified total RNA using a QuantiTect® Reverse Transcription Kit (Qiagen). The PCR reaction was performed in a thermal cycler of ProFlex PCR system (Applied Biosystems, Foster City, CA) by PCR amplification using *Ex Taq*® DNA polymerase (Takara, Tokyo, Japan) under the reported conditions [26]. Two months after the grafting, fresh leaves were sampled to check for CTV infection in the scion of the BC_2_ null segregants using ImmunoStrip® for Citrus tristeza virus (CTV) Complete kit (Agdia, IN, USA) under the manufacture’s direction. The scions of ‘Hyuganatsua’ and trifoliate orange were used for susceptible control and resistant control, respectively.

## Supplementary information

**Additional file 1: Table S1.** CAPS marker information used in the genetic linkage map and graphical genotyping map.

**Additional file 2: Fig. S1.** The position of T-DNA integrated region on the trifoliate genome assembles visualized by JBrowser in Mikan Genome Database (MiGD:https://mikan.dna.affrc.go.jp/).

## Data Availability

The complete CGH analysis data are deposited in the NCBI Gene Expression Omnibus (http://www.ncbi.nlm.nih.gov/geo/) under series entry GSE135027.

## Abbreviations

BC: Backcross
CAPS: Cleaved amplified polymorphic sequences
CGH: Comparative genome hybridization
CTV: Citrus tristeza virus
LG: Linkage group
MAS: Marker-assisted selection
NGS: Next-generation sequencing
